# Bioimpedance Sensor Array for Long-Term Monitoring of Wound Healing from Beneath the Primary Dressings and Controlled Formation of H_2_O_2_ Using Low-Intensity Direct Current

**DOI:** 10.3390/s19112505

**Published:** 2019-05-31

**Authors:** Atte Kekonen, Mikael Bergelin, Max Johansson, Narender Kumar Joon, Johan Bobacka, Jari Viik

**Affiliations:** 1 Faculty of Medicine and Health Technology, Tampere University, Korkeakoulunkatu 3, FI-33720 Tampere, Finland; jari.viik@tuni.fi; 2 Turku PET Centre, Åbo Akademi Accelerator Laboratory, c/o Turku University Hospital, Kiinamyllynkatu 4-8, FI-20520 Turku, Finland; mikael.bergelin@abo.fi; 3 CutoSense Ltd., Kaarinantie 700, FI-20540 Turku, Finland; max.johansson@cutosense.fi; 4 Laboratory of Analytical Chemistry, Johan Gadolin Process Chemistry Centre, Åbo Akademi University, Biskopsgatan 8, FI-20500 Turku, Finland; narender.joon@abo.fi (N.K.J.); johan.bobacka@abo.fi (J.B.)

**Keywords:** bioimpedance, quasi-monopolar, wound monitoring, multielectrode, sensor array, wound dressing, long-term monitoring, beneath the dressings, hydrogen peroxide, wound stimulation, low-intensity direct current

## Abstract

Chronic wounds impose a significant financial burden for the healthcare system. Currently, assessment and monitoring of hard-to-heal wounds are often based on visual means and measuring the size of the wound. The primary wound dressings must be removed before assessment can be done. We have developed a quasi-monopolar bioimpedance-measurement-based method and a measurement system to determine the status of wound healing. The objective of this study was to demonstrate that with an appropriate setup, long-term monitoring of wound healing from beneath the primary dressings is feasible. The developed multielectrode sensor array was applied on the wound area and left under the primary dressings for 142 h. The impedance of the wounds and the surrounding intact skin area was measured regularly during the study at 150 Hz, 300 Hz, 1 kHz, and 5 kHz frequencies. At the end of the follow-up period, the wound impedance had reached the impedance of the intact skin at the higher frequencies and increased significantly at the lowest frequencies. The measurement frequency affected the measurement sensitivity in wound monitoring. The skin impedance remained stable over the measurement period. The sensor array also enabled the administration of periodical low-intensity direct current (LIDC) stimulation in order to create an antimicrobial environment across the wound area via the controlled formation of hydrogen peroxide (H_2_O_2_).

## 1. Introduction

A wound is defined as an interruption in the continuity of a body structure, especially an injury in which the protective layer such as the skin is damaged [1]. A wound is ambiguously defined as chronic if it fails to heal in timely manner through normal tissue repair processes despite active treatment [2]. In most cases, a nonhealing wound is the result of a systemic disease such as impairment of venous or arterial circulation or metabolic syndrome. Also, bed-ridden patients are prone for chronic ulceration.

Chronic wounds impose a significant and increasing burden to the healthcare system. In the United States alone (in 2009), chronic wounds affected 6.5 million patients, and in excess of US$25 billion is spent annually on the treatment of chronic wounds [3]. In Europe, around 1.5–2 million patients suffer from chronic wounds. It is suggested that 64% of wounds treated in homecare in Europe are of chronic etiology [4]. An aging population, diabetes, and lifestyle-related issues such as metabolic syndrome and obesity increase the prevalence of hard-to-heal wounds. Venous ulcers account for 70–90% of ulcers found on the lower leg. Treatment of a venous leg ulcer costs circa US$9600 on average [3]. In most cases, venous leg ulcer patients are treated at home along with regular or occasional visits to outpatient clinics or specialized wound centers [5].

Before a chronic wound can be expected to heal, the systemic factors have to be brought under control. For example, a patient’s nutritional status and medication has to be inspected and smoking must cease [6]. Patients with chronic venous insufficiency suffer from sustained venous hypertension, which eventually leads to lower-extremity edema. Lower-extremity edema contributes to ulceration in many ways and significantly impairs healing of venous ulcers. The backbone of treatment of a venous ulcer is compression therapy, which reduces venous stasis and swelling [7]. Local wound care includes debridement and cleansing of the wound bed. Choosing an appropriate wound dressing is important. Venous ulcers are typically moist and absorbing primary dressings are used. Sometimes, venous surgery is necessary to correct vascular impairment; also, skin grafting may be applied [8,9].

Assessment and monitoring of chronic wounds in clinical practice is often based on visual evaluations of variables such as the color of the wound bed, the amount and color of the exudate and the debris, and the odor and overall condition of the surrounding skin. The wound size is often measured [10]. These methods are either subjective or require the removal of the primary dressings.

Impedance, which is the ratio between voltage and current, is a frequency-dependent variable and describes the ability of a material to oppose the flow of alternating electrical current. Bioimpedance describes the passive electrical properties of biological tissues [11]. Bioimpedance measurement has been previously used in dermatological research, for example, to evaluate the hydration status of the skin, for skin cancer diagnosis, and to measure transdermal drug delivery [12,13,14]. Bioimpedance measurement has also been applied to evaluate wound healing. Lukaski et al. (2012) studied wound healing using bioimpedance measurement in a tetrapolar arrangement [15]. They monitored wounds of various statuses for several weeks in a noncontinuous manner with promising results. Swisher et al. (2015) introduced a smart bandage for early detection of pressure-induced tissue damage in a rat model using bipolar bioimpedance measurement in vivo [16]. Also, other related studies have been published in recent years [17,18,19,20].

Hydrogen peroxide is a well-known bactericide. It is naturally produced in cellular processes during wound healing [21]. Historically, it has been applied topically as an antiseptic for cleansing wounds. Presently, the topical use of high-concentration H_2_O_2_ (typically 3%) has been reduced as it is nonspecific and also kills protagonist cells in the wound base. However, recently, several studies have suggested that long-term low-concentration exposure to H_2_O_2_ would be beneficial for wound healing. Studies have also suggested that long-term low-concentration exposure to H_2_O_2_ destroys biofilms, including methicillin-resistant biofilms [22,23]. Reports by Sultana et al. (2015) and more recently Raval et al. (2019) have suggested that low-stimulation voltages produce low but clinically significant concentrations of H_2_O_2_ [22,23].

We have previously introduced a bioimpedance-measurement-based method and an early prototype of a measurement system for monitoring wound healing [17,18,24]. We have shown that the bioimpedance method is a promising tool for monitoring acute and chronic wound healing in noncontinuous measurements [25,26]. In this study, we introduced a new prototype of a multielectrode sensor array for long-term bioimpedance monitoring of wound healing from beneath the primary wound dressings. The objective of this study was to evaluate whether it is possible to continuously monitor wound healing from beneath the primary wound dressing for an extended period of time with our measurement setup. We also studied the long-term behavior of skin impedance and the effect of measurement frequency on wound monitoring sensitivity and the stability of the skin impedance. Additionally, we arranged a laboratory test to study if it is possible to produce H_2_O_2_ using low-intensity direct current (LIDC) stimulation with our electrode setup.

## 2. Materials and Methods

### 2.1. Measurement Instrumentation

The bioimpedance measurement instrumentation consisted of a perforated multielectrode sensor array and a purpose-built bioimpedance measurement system for wound monitoring. The prototype of the bioimpedance measurement system was introduced by Kekonen et al. (2016) [18]. The sensor array was based on the design presented by Kekonen et al. (2018) [24].

#### 2.1.1. Sensor Array

**Figure 1:**
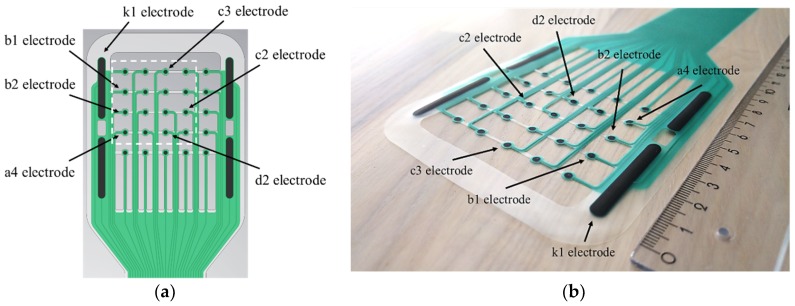
(**a**) The layout of the multielectrode sensor array prototype. (**b**) A photograph of the sensor array. The dashed white square marks the electrodes which participated in the measurements. The electrodes k1, a4, b1, b2, c2, c3, and d2 are marked on the figure. The electrodes a4, b1, and b2 were on the wound in the first experiment and the electrodes c2, c3, and d2 in the second experiment. The remaining electrodes in the array and on the left and the right side of the array were on the intact skin. The sensor array was based on the design presented by Kekonen et al. (2018) [24].

The new prototype of the sensor array (Figure 1) was based on a thin thermoplastic polyurethane (TPU) substrate. TPU is a soft, malleable, and slightly stretchable material. The overall size of the electrode head was 95 × 100 mm, and the size of the tail section was 40–50 × 400 mm. The sensor array contained an array of 25 silk-screen-printed circular electrodes with a diameter of 2 mm each, encircled by 4 counter electrodes with dimensions of 4 × 35 mm. The electrodes in the array were 12 mm apart. The electrodes and the leads consisted of electrically conductive silver-ink, and the electrode surfaces were coated with a thin layer of biomedical-grade carbon ink. The 500 µm width leads were electrically insulated with a layer of dielectric; thus, only the contact surfaces of the electrodes were exposed to the tissue. The excess substrate material was removed, so that the free space was maximized and the path for the wound exudate and moisture to transport to the primary dressings was open.

#### 2.1.2. Bioimpedance Measurement System

The bioimpedance measurement system consisted of a measurement circuit for multifrequency bioimpedance measurement. It also included multiplexer blocks, so that the two-electrode measurements can be performed either using the predetermined measurement sequence or, if desired, the electrode pairs can be selected by the user individually. The device transmitted the measurement data via a Bluetooth link to the graphical user interface on a PC, which performed the initial calculation and visualization of the results.

### 2.2. The Skin and Wound Impedance Monitoring Study

We arranged two experiments with similar setups to examine the feasibility of the multielectrode sensor array for long-term monitoring of wound healing from beneath the primary dressings. In these experiments, we also studied the behavior of the skin impedance. The impedance was measured in a quasi-monopolar configuration. The small electrodes, named a1 to d4, acted as the active electrodes, whereas the larger k1 electrode worked as the counter electrode. We also measured skin impedance in a bipolar configuration using the equally sized larger electrodes k1 and k2.

During the 142 h long follow-up, the subject lived a normal life without restrictions. The subject did light exercise during the follow-up and, when showering, the dressings were protected with a waterproof plastic cover.

#### 2.2.1. Measurement Arrangement for the Impedance Measurements

**Figure 2:**
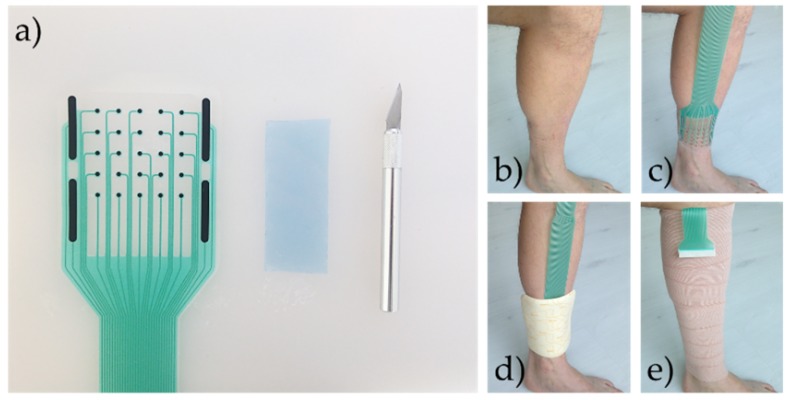
(**a**) The materials needed for preparing the multielectrode sensor array before placement on the skin. (**b**,**c**) The placement of the sensor array on the left shin. (**d**) A foam dressing applied on top of the sensor array. (**e**) Compression bandage folded on the shin; only the connector end was outside of the bandage.

In the first experiment, we monitored the healing of three small acute cut wounds from beneath the primary dressings. The sensor array was prepared as shown in the Figure 2a by placing circa 2 mm diameter and 1 mm thick hydrogel pads on the circular electrodes of the array and circa 4 × 25 mm pads on the counter electrodes surrounding the array. The sensor array was placed on the intact skin of the left shin (Figure 2b,c). A Biatain™ foam dressing was placed on top of the sensor array, tubular gauze fabric was placed under the tail section of the sensor array, and finally, a compression bandage was folded around the leg (Figure 2d,e). We measured the intact skin impedance for the first 24 h. Then, the compression bandage was carefully removed and the foam dressing slightly lifted in a way that three wounds could be induced using a surgical lancet under the electrodes a4, b1, and b2. After this, the dressings were reapplied. The procedure took less than 5 min. The wound was circa 3 mm in length, extended to the dermis, and was bleeding slightly. The skin condition around the measurement area was dryish and flaky but intact. The wound and the skin impedances were measured at 150 Hz, 300 Hz, 1 kHz, and 5 kHz frequencies in two electrode configurations from multiple locations according to the electrodes of the array, so that the counter electrode was k1. The wound and the skin impedances from beneath the primary dressings were monitored for a total of 142 h.

In the second experiment, we reproduced the arrangement of the first experiment for the right shin. The skin condition was normal in the area of measurement. After 24 h of monitoring the skin impedance, three small acute wounds were induced on the skin using a surgical lancet under the electrodes c2, c3, and d2. The wounds were similar in size and depth as in the first study. The foam dressing and the compression bandage were reapplied immediately after inducing the wounds. The counter electrode in all measurements was k1.

During the entire 142 h follow-up period, there were 40 measurement events in the first experiment and 31 measurement events in the second experiment. In each measurement event, the measurements were performed using a total of 17 electrode pairs. Of these, three electrode pairs were occupied by the wound impedance measurement (after inducing the wounds), 13 electrode pairs measured the skin impedance, and additionally, one electrode pair with a larger electrode surface area measured the skin impedance (k1–k2).

#### 2.2.2. Impedance Data Analysis

We calculated the average skin impedance and the standard deviation for each individual skin impedance measuring electrode pair and each measurement frequency (150 Hz, 300 Hz, 1 kHz, and 5 kHz) between 24 and 142 h. Between 24 and 142 h, there were 29 measurement events in the first experiment and 22 in the second experiment. We also calculated the average skin impedance and the average of the standard deviation of all 13 skin impedance measuring electrode pairs between 24 and 142 h. We referred to these variables as the total average skin impedance ($\bar{Z}$_total_) and the total standard deviation (σ_total_). Additionally, we calculated the standard deviation (σ) of the total average skin impedance.

We also calculated the average skin impedance ($\bar{Z}$) and the standard deviation (σ) for both experiments at 150 Hz, 300 Hz, 1 kHz, and 5 kHz frequencies just before and right after inducing the wounds on the skin underneath the three electrode pairs.

### 2.3. Hydrogen Peroxide Measurements

Periodic LIDC stimulation can be administered to a wound by utilizing the circular electrodes in parallel-connected form as a cathode and parallel-connected counter electrodes as an anode. Our primary interest in using LIDC was the possibility to create an antimicrobial environment across the wound area via controlled formation of H_2_O_2_ on the cathode electrode surfaces. It has been shown that a sufficiently low H_2_O_2_ concentration can destroy biofilm and maintain an antimicrobial wound environment, without significant adverse effects on cell proliferation [22,23]. By utilizing LIDC at a set potential of 1.3 V, which is slightly above the thermodynamic stability of the water in intercellular fluid, a manageable amount of H_2_O_2_ is formed via the reaction between water and oxygen radicals at the cathode surface. Using LIDC in periodical mode allows the formed H_2_O_2_ to dissipate into the intercellular fluid volume between polarization pulses, thereby ensuring the desired µM-level concentration of H_2_O_2_ and sought-after effect.

A separate laboratory test was performed in order to verify the formation of H_2_O_2_ on the screen-printed carbon cathodes of the sensor array. The concentration of electrochemically generated H_2_O_2_ was determined amperometrically by reduction of the formed H_2_O_2_ at a screen-printed Prussian-blue-modified electrode. For this purpose, a laboratory-built Teflon measurement cell was constructed, which was mounted on top of the carbon electrode prints with a 1.25 mm gap between the carbon ink cathode and a screen-printed Prussian-blue-modified electrode (DRP-710, DropSens) that was used to detect H_2_O_2_ amperometrically. Prior to the measurement, the Prussian-blue-coated DropSens electrode was calibrated by spiking known quantities of 0.1 M H_2_O_2_ solution (EMSURE, 30%) into 20 mL of phosphate-buffered saline (PBS) solution (NaCl—8.0 g; Na_2_HPO_4_—1.44 g; KCl—0.2 g; KH_2_PO_4_·3H_2_O—0.24 g, standard addition method). The current was measured at −0.1 V. The sensitivity of the DropSens electrode was found to be excellent in the micromolar range, and the slope was found to be −4 nA/µM. Prior to the analysis, the DropSens electrode was conditioned in PBS for 15 min to achieve a faster steady state. The cell was assembled, the printed carbon ink electrodes were connected to an EmStat potentiostat (PalmSens), and the potential (1.3 V) was applied between anode and cathode prints. The formation of H_2_O_2_ was detected with the DropSens electrode connected to a second EmStat potentiostat.

## 3. Results

### 3.1. The Skin and Wound Impedance Measurements

**Figure 3:**
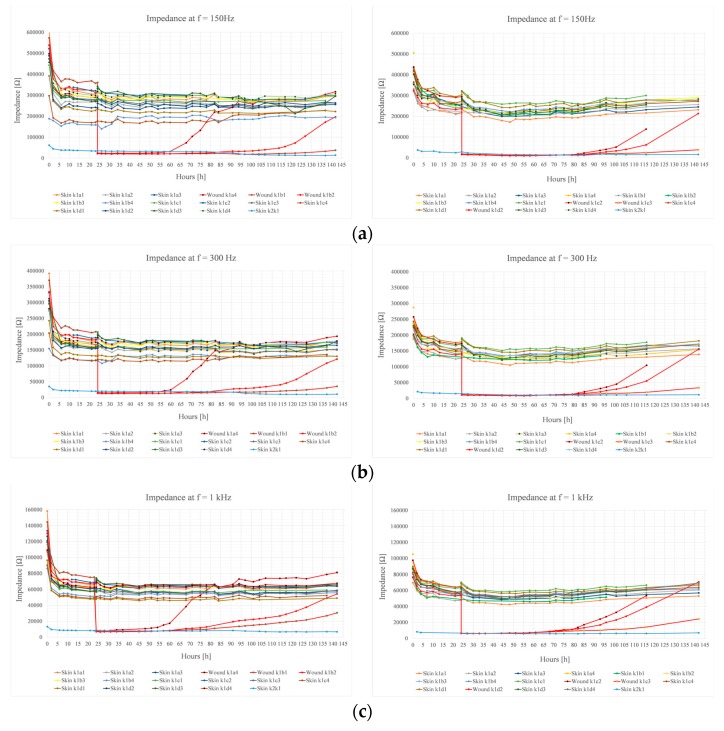

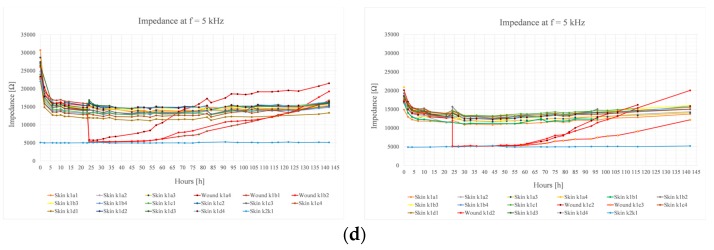
The impedance measurement results of the first experiment (left column) and the second experiment (right column) for 150 Hz, 300 Hz, 1 kHz, and 5 kHz frequencies. In both experiments, the impedance was measured from beneath the primary dressings for 142 h. A total of 17 electrode pairs were used in the measurements. In the first experiment, a wound was induced under the electrodes a4, b1, and b2 after 24 h of measuring skin impedance. In the second experiment, a wound was induced under the electrodes c2, c3, and d2 after 24 h of measuring skin impedance. The remaining 14 electrode pairs measured skin impedance over the 142 h follow-up period. The electrode pair k1–k2 consisted of electrodes which had a larger surface area. (**a**) 150 Hz; (**b**) 300 Hz; (**c**) 1 kHz; (**d**) 5 kHz.

Figure 3 represents the results of the first and second experiments. The protocol of the first experiment was reproduced in the second experiment. The impedance of three wounds and intact skin was measured from multiple locations at 150 Hz, 300 Hz, 1 kHz, and 5 kHz frequencies. After 24 h of measuring skin impedance, the wound dressings were carefully removed and wounds were induced under electrodes a4, b1, and b2 in the first experiment. In the second experiment, the wounds were induced under electrodes c2, c3, and d2. The wound dressings were then reapplied. The remaining electrodes measured skin impedance over the whole 142 h follow-up period. The electrode k1 was the counter electrode in all measurements. The electrode k2 had a larger surface area, similar to k1, and was on intact skin.

Table 1 represents the calculated skin impedance variables of both experiments. The variables include the total average skin impedance ($\bar{Z}$_total_), the total standard deviation (σ_total_), and the standard deviation (σ) of the total average skin impedance. The results are based on the skin impedance measurements between 24 and 142 h of 13 electrode pairs.

Table 2 depicts the calculated impedance variables of both experiments just before and immediately after inducing the wounds. In the first experiment, the wounds were induced under electrodes a4, b1, and b2 after 24 h of measuring skin impedance. In the second experiment, this was done under the electrodes c2, c3, and d2. An immediate and significant decrease of impedance was observed in all measurement frequencies in both experiments after inducing the wounds. In the first experiment, results could not be obtained at 1 and 5 kHz at this time due temporary problems with the electrode leads.

**Figure 4:**
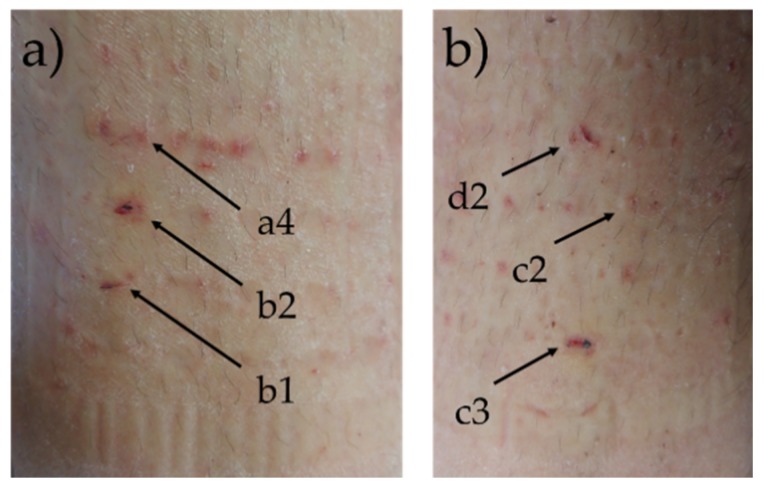
The condition of the skin and the wounds after 142 h of bioimpedance follow-up. The location of the wound electrodes is marked in the photos. (**a**) The first experiment. (**b**) The second experiment.

In the first experiment, the first signs of modest but conclusive impedance increase of electrode pair k1–a4 could be seen 2–4 h after inducing the wounds at 5 kHz frequency (Figure 3, left column). The time difference between the first signs of impedance increase at 150 Hz and 5 kHz frequencies was approximately 24 h. In all frequencies, the slope of the impedance magnitude gradually increased. The slope decreased when the wound impedance reached or exceeded the level of intact skin impedance. After 142 h of follow-up, the remaining leads cut off. The wound impedances at 5 kHz had surpassed the general level of the intact skin impedance. At 1 kHz frequency, the wound impedance measuring electrode pair k1–a4 had exceeded the average skin impedance, electrode pair k1–b2 had reached the average skin impedance, and electrode pair k1–b1 had not yet reached the average skin impedance. At 300 and 150 Hz, electrode pair k1–a4 had surpassed the average skin impedance, the electrode pair k1–b1 was closing the average skin impedance, and the electrode pair k1–b2 had not yet reached the average skin impedance. The wound dressings were removed after 142 h follow-up. By visual evaluation, it was noted that the wounds under electrodes a4 and b1 had re-epithelized, while the tissue under electrode b2 did not appear to be completely re-epithelized (Figure 4a). However, it is unclear if the wound had slightly reopened during removal of the dressings.

In the second experiment, the first conclusive signs of impedance increase of electrode pairs k1–d2 and k1–c2 were noticed at 5 kHz frequency circa 20 h after inducing the wounds (Figure 3, right column). The impedance first increased at the higher measurement frequencies, which occurred in all three wound electrode pairs. The time difference between the first signs of impedance increase at 150 Hz and 5 kHz frequencies was approximately 14 h. The wound impedance increased in an accelerating manner, and the slope of impedance magnitude increased. The wound impedances did not reach the level of the skin impedance at 150 Hz by the end of the follow-up period, and additionally, we were not able obtain measurement results from k1–c2 after 116 h. However, the trend clearly showed that the wound impedances were closing to the level of the skin impedances. At higher frequencies, the impedance of wound measuring electrode pair k1–d2 exceeded the level of the skin impedances by the end of the follow-up period. The electrode pair k1–c2 reached the level of skin impedance at higher frequencies by 116 h, after which we could not obtain any further results, as the electrode leads had cut off. The impedance of wound measuring electrode pair k1–c1 also seemed to close in and almost reach the level of the intact skin impedances by the end of the follow-up 142 h from the start of the experiment. After 142 h, the remaining electrode leads cut off and no further measurement results could be obtained. The wound dressings were removed after 142 h of impedance follow-up. By visual evaluation, it was noted that the wounds under electrodes c2 and d2 had re-epithelized, while the tissue under electrode c3 did not appear to be completely re-epithelized (Figure 4b). However, also in this case, it was unclear whether the wound had actually slightly reopened during removal of the dressings.

### 3.2. Hydrogen Peroxide Measurement Results

The formation of H_2_O_2_ was studied separately in a small-volume measurement cell. While analyzing, to minimize the noise, the curves were smoothened out (20 points). To determine the H_2_O_2_ concentration, a baseline current was obtained from the last 2–3 min from each part (just prior to the polarization) and the first current value after the polarization was used to determine the H_2_O_2_ concentration. For the concentration calculation, a slope of −4 nA/μM was used as determined by the calibrations. It was found that with a polarization time of 60 s, the H_2_O_2_ concentration was 33 µM in the vicinity of the cathode and leveled out at 65 µM when the polarizing time exceeded 120 s (tested up to 300 s). Results are shown in Table 3.

## 4. Discussion

In this article, we have introduced a prototype of a multielectrode sensor array. We have successfully demonstrated the feasibility of the sensor array for long-term monitoring of intact skin and wound healing from beneath the primary dressings. To the best of our knowledge, this is the first time the bioimpedance method has been used successfully to monitor wound healing continuously for a long time under difficult conditions. We have also shown that the multielectrode sensor array with LIDC stimulation produces clinically relevant concentrations of H_2_O_2_ in order to form an antibacterial environment around the wound area.

The intact skin and wound impedances were monitored for 142 h from beneath the primary dressings in two experiments with the same setup. The skin impedance in both experiments remained very stable throughout the 142 h follow-up period, regardless of light exercise and other normal life activities. The wound impedance increased first at the higher frequencies. By the end of the 142 h follow-up period, the wound impedances had either reached, exceeded, or significantly increased to match the skin impedance in all measured frequencies.

The level of skin impedance, especially at the lower measurement frequencies, depends on the moisture level of the skin, thickness of the skin, and physiological state of the skin, among other factors. It is known that continuously (at-line) measuring skin impedance in a reliable and stable fashion for extended periods of time is very difficult. Our results show that with an appropriate setup, it is feasible, even in relatively difficult conditions. Our results also indicate that higher stability of skin impedance as a function of time can be achieved at higher measurement frequencies. However, there is a trade-off between the magnitude level of the skin impedance and the higher measurement frequency. The magnitude level of skin impedance decreases steeply with increasing measurement frequency.

An initial decrease of intact skin impedance in the first hours of the experiments was observed in all measurement frequencies. The initial decrease was largest, proportionally and as absolute values, at the lowest frequencies. This was most likely due to the skin absorbing the moisture delivered by the hydrogel pads, which had the largest effect on the superficial layers of the skin. The small hydrogel pads provided very good mildly adhesive contact with the skin and advantageously balanced the moisture conditions. Excessive dryness of the skin was avoided by the hydrogel releasing moisture to the skin. In the first experiment, the skin condition was clearly distinct from the second experiment. The skin in the first experiment was dry and flaky. In the second experiment, the skin condition was normal. Regardless of these differences, the intact skin impedances in both experiments were at the same level. The hydrogel also absorbed the blood and therefore prevented the formation of a dry scab; presumably, this also applied for the wound exudate. A moist wound environment and prevention of scab formation has been shown to be beneficial for wound healing [27].

The electrode surface area and conductive materials, the electrode–skin contact area, the electrolyte composition, and the measurement frequency are key factors for optimizing two-electrode bioimpedance measurements for different applications. The effect of electrode–skin contact area can be clearly seen from results when comparing the impedance magnitude levels measured by the electrode pair k1–k2 with larger and equal electrode surface areas (bipolar measurement) and other electrode pairs (quasi-monopolar measurement). The larger the electrode–skin contact area, the smaller the impedance magnitude. The quasi-monopolar configuration is essential for targeting the sensitivity to the area of interest. In the quasi-monopolar configuration, the active electrode, which has the smaller skin-contact area, provides higher impedance contribution to total impedance due to the straight-forward relationship between current density and sensitivity of the two-electrode bioimpedance setup. We have not found the electrode impedance to be a significant debilitating factor for bioimpedance measurements at these quite low frequencies, where the skin impedance provides a significant proportional contribution to the total impedance.

In both experiments, the wounds induced a drastic and immediate reduction of the measured impedance. The impedance reduction was mostly the result of a breach in the stratum corneum layer of the skin and the break-up of the other epidermal layers. The stratum corneum is known to provide a barrier against external agents such as chemicals, pathogens, and electrical current [28]. In both experiments, the increase in wound impedance was first observed at higher frequencies a few hours after inducing the wounds. At lower frequencies, the recovery of the impedance started later. It seems that regardless of the measurement frequency, the impedance increased modestly at first, followed by a phase of rapid increase. The latter phase may have been due to wound contraction and the formation of the neo-epithelium on the surface of the wound, which we have observed in earlier studies to have a significant impact on the wound impedance [25,26]. At the end of the 142 h follow-up period, the wound impedance at higher frequencies had reached or exceeded the level of the skin impedance. At 150 Hz frequency, the impedance of all wound measuring electrodes had increased significantly and some had reached the impedance of the skin.

We tested the production of H_2_O_2_ using LIDC stimulation. A 1.3 V polarization at a duration of 120 s between measurement and counter electrodes yielded a H_2_O_2_ concentration of 65 µM. Due to the limited signal-to-noise ratio in the measured currents, precise concentrations cannot be guaranteed and should be treated with caution. However, it is clear that H_2_O_2_ was formed on the printed carbon ink cathodes, in the desired concentration range, and was detectable in the PBS solution at a significant distance (1.25 mm) from the surface of the cathode electrode.

## 5. Conclusions

In this study, we have demonstrated that the multielectrode sensor array is capable of long-term monitoring of intact skin and acute wound healing from the beneath the primary dressings. The results indicated that by an appropriate selection of measurement frequency, it is possible to improve the sensitivity of the wound measurement, particularly in the early phase of the healing process, or to optimize the sensitivity to unveil the characteristics of different skin layers or tissue depths. It was also found that a clinically relevant amount of H_2_O_2_ could be produced by polarization of electrodes. This H_2_O_2_ concentration participates in the production of an antimicrobial environment around the wound area.

## Figures and Tables

**Table 1:** The calculated skin impedance variables of the first and second experiments between 24 and 142 h of follow-up.

f	Experiment 1			Experiment 2		
	$\bar{Z}$_total_	σ_total_	σ	$\bar{Z}$_total_	σ_total_	σ
150 Hz	256.0 kΩ	11.2 kΩ	35.8 kΩ	233.0 kΩ	14.3 kΩ	19.6 kΩ
300 Hz	153.8 kΩ	5.1 kΩ	17.4 kΩ	140.2 kΩ	8.6 kΩ	11.7 kΩ
1 kHz	58.6 kΩ	1.3 kΩ	5.7 kΩ	54.1 kΩ	3.0 kΩ	4.2 kΩ
5 kHz	14.1 kΩ	0.5 kΩ	1.0 kΩ	13.0 kΩ	0.7 kΩ	0.7 kΩ

**Table 2:** The calculated impedance variables of the first experiment and second experiments before and after inducing the wounds. The skin impedance results at 1 and 5 kHz in the first experiment could not be obtained due to temporary problems with the electrode leads.

f	Experiment 1				Experiment 2			
	Before Wounding (Skin)		After Wounding (Wounds)		Before Wounding (Skin)		After Wounding (Wounds)	
	$\bar{Z}$	σ	$\bar{Z}$	σ	$\bar{Z}$	σ	$\bar{Z}$	σ
150 Hz	323.9 kΩ	27.3 kΩ	22.8 kΩ	1.1 kΩ	265.6 kΩ	23.5 kΩ	17.0 kΩ	1.0 kΩ
300 Hz	186.2 kΩ	16.2 kΩ	14.7 kΩ	1.0 kΩ	157.7 kΩ	13.7 kΩ	11.6 kΩ	0.7 kΩ
1 kHz			7.9 kΩ	0.8 kΩ	58.7 kΩ	4.5 kΩ	6.3 kΩ	0.2 kΩ
5 kHz			5.5 kΩ	0.3 kΩ	13.6 kΩ	0.6 kΩ	5.1 kΩ	0.1 kΩ

**Table 3:** Hydrogen peroxide concentration as a function of polarization time in phosphate-buffered saline (PBS), 1.25 mm from cathode surface.

Polarization Time	Baseline Current (Prior to Polarization)	Current (After Polarization)	H_2_O_2_ Concentration
60 s (200–260)	−1.964 µA	−2.095 µA	33 µM
120 s (400–520)	−1.867 µA	−2.126 µA	65 µM
300 s (800–1100)	−1.842 µA	−2.093 µA	63 µM

## References

[1] Stedman T.L. (2004). The American Heritage Stedman’s Medical Dictionary.

[2] Kirsner R.S., Vivas A.C. (2015). Lower-extremity ulcers: Diagnosis and management. Br. J. Dermatol..

[3] Sen C.K., Gordillo G.M., Roy S., Kirsner R., Lambert L., Hunt T.K., Gottrup F., Gurtner G.C., Longaker M.T. (2009). Human skin wounds: A major and snowballing threat to public health and the economy. Wound Repair Regen..

[4] Lindholm C., Searle R. (2016). Wound management for the 21st century: Combining effectiveness and efficiency. Int. Wound J..

[5] Rondas A.A.L.M., Schols J.M.G., Halfens R.J.G., Hull H.R., Stobberingh E.E., Evers S.M.A.A. (2015). Cost analysis of one of the first outpatient wound clinics in the Netherlands. J. Wound Care.

[6] Panuncialman J., Falanga V. (2007). The science of wound bed preparation. Clin. Plast. Surg..

[7] Simon D.A., Dix F.P., McCollum C.N. (2004). Management of venous leg ulcers. BMJ.

[8] Obermayer A., Göstl K., Walli G., Benesch T. (2006). Chronic venous leg ulcers benefit from surgery: Long-term results from 173 legs. J. Vasc. Surg..

[9] Jones J.E., Nelson E.A., Al-Hity A. (2013). Skin grafting for venous leg ulcers. Cochrane Database Syst. Rev..

[10] Grey J.E., Enoch S., Harding K.G. (2006). ABC of wound healing: Wound assessment. BMJ.

[11] Grimnes S., Martinsen O. (2015). Bioimpedance and Bioelectricity Basics.

[12] Tagami H., Ohi M., Iwatsuki K., Kanamaru Y., Yamada M., Ichijo B. (1980). Evaluation of skin surface hydration in vivo by electrical measurement. J. Invest. Dermatol..

[13] Aberg P., Nicander I., Hansson J., Geladi P., Holmgren U., Ollmar S. (2004). Skin cancer identification using multifrequency electrical impedance: A Potential screening tool. IEEE Trans. Biomed. Eng..

[14] Arpaia P., Cesaro U., Moccaldi N. (2017). Noninvasive measurement of transdermal drug delivery by impedance spectroscopy. Sci. Rep..

[15] Lukaski H.C., Moore M. (2012). Bioelectrical impedance assessment of wound healing. J. Diabetes Sci. Technol..

[16] Swisher S.L., Lin M.C., Liao A., Leeflang E.J., Khan Y., Pavinatto F.J., Mann K., Naujokas A., Young D., Roy S. (2015). Impedance sensing device enables early detection of pressure ulcers in vivo. Nat. Commun..

[17] Kekonen A., Bergelin M., Eriksson J.-E., Ylänen H., Viik J. (2015). A quantitative method for monitoring wound healing. Int. J. Bioelectromagn..

[18] Kekonen A., Bergelin M., Eriksson J.-E., Ylänen H., Kielosto S., Viik J. (2016). Bioimpedance Measurement System for Evaluation of the Status of Wound Healing. Proceedings of the 15th Biennial Baltic Electronics Conference.

[19] Mehmood N., Hariz A., Templeton S., Voelcher N.H. (2014). A flexible and low power telemetric sensing and monitoring system for chronic wound diagnostics. Biomed. Eng. Online.

[20] Moore Z., Patton D., Rhodes S.L., O’Connor T. (2017). Subepidermal moisture (SEM) and bioimpedance: A literature review of a novel method for early detection of pressure-induced tissue damage (pressure ulcers). Int. Wound J..

[21] Zhu G., Wang Q., Lu S., Niu Y. (2017). Hydrogen peroxide: A potential wound therapeutic target?. Med. Princ. Pract..

[22] Sultana S.T., Atci E., Babauta J.T., Falghoust A.M., Snekvik K.R., Call D.R., Beyenal H. (2015). Electrochemical scaffold generates localized, low concentration of hydrogen peroxide that inhibits bacterial pathogens and biofilms. Sci. Rep..

[23] Raval Y.S., Mohamed A., Zmuda H.M., Patel R., Beyenal H. (2019). Hydrogen-peroxide-generating electrochemical scaffold eradicates methicillin-resistant *Staphylococcus aureus* biofilms. Glob. Chall..

[24] Kekonen A., Bergelin M., Eriksson J.-E., Vesa M., Johansson M., Viik J. (2018). Long-Term Monitoring of Acute Wound Healing from Beneath the Primary Wound Dressings. Proceedings of the 16th Biennial Baltic Electronics Conference.

[25] Kekonen A., Bergelin M., Eriksson J.-E., Vaalasti A., Ylänen H., Viik J. (2017). Bioimpedance measurement based evaluation of wound healing. Physiol. Meas..

[26] Kekonen A., Bergelin M., Eriksson J.-E., Vaalasti A., Viik J. Proof-of-Concept Study for Bioimpedance Based Monitoring of Venous Ulcers during Galvanic Stimulation. https://clinicaltrials.gov/ct2/show/NCT02101645.

[27] Hess C. (2011). Checklist for factors affecting wound healing. Adv. Skin Wound Care.

[28] Bouwstra J.A., Ponec M. (2006). The skin barrier in healthy and diseased state. Biochim. Biophys. Acta.

